# Supplementary material for: Comparative Analysis of Subclassification Systems in Patients with Intermediate-Stage Hepatocellular Carcinoma (Barcelona Clinic Liver Classification B) Receiving Systemic Therapy
Source: Curr Oncol. 2024 Jan 19;31(1):547–57. doi: 10.3390/curroncol31010038 (PMC10814359; doi:10.3390/curroncol31010038)
Supplement: Supplementary file 1 [file curroncol-31-00038-s001.zip › curroncol-2767403-supplementary.pdf]

Supplementary Table 1. Stratification of overall survival according to the subclassification systems.

Bolondi system according to AFP	n	OS, mo (95%CI)	HR (95%CI)	*p*	HR (95%CI)	*p*	HR (95%CI)	*p*	HR (95%CI)	*p*	HR (95%CI)	*p*
B1 <400	38 (22.2%)	22.5 (17.3-27.8)	Ref.									
B1 >400	8 (4.7%)	15.2 (0.1-47.9)	1.011 (0.392-2.607)	0.983	Ref.	-						
B2 <400	81 (47.7%)	15.2 (8.4-21.9)	1.712 (1.123-2.610)	0.012	1.701 (0.686-4.217)	0.252	Ref.	-	-			
B2 >400	33 (19.3%)	7.0 (5.5-8.5)	3.416 (2.061-5.660)	<0.001	3.410 (1.320-8.808)	0.011	2.014 (1.324-3.063)	0.001	Ref.	-	-	
B3 <400	6 (3.5%)	6.6 (0.1-16.8)	2.893 (1.196-7.000)	0.018	2.888 (0.876-9.522)	0.081	1.695 (0.735-3.907)	0.216	0.885 (0.366-2.140)	0.787	Ref.	-
B3 >400	5 (2.9%)	4.4 (1.9-6.9)	11.685 (4.303-31.731)	<0.001	11.505 (3.189-41.506)	<0.001	6.693 (2.565-17.466)	<0.001	2.479 (0.915-6.671)	0.074	2.729 (0.636-11.707)	0.177

AFP: alpha-fetoprotein

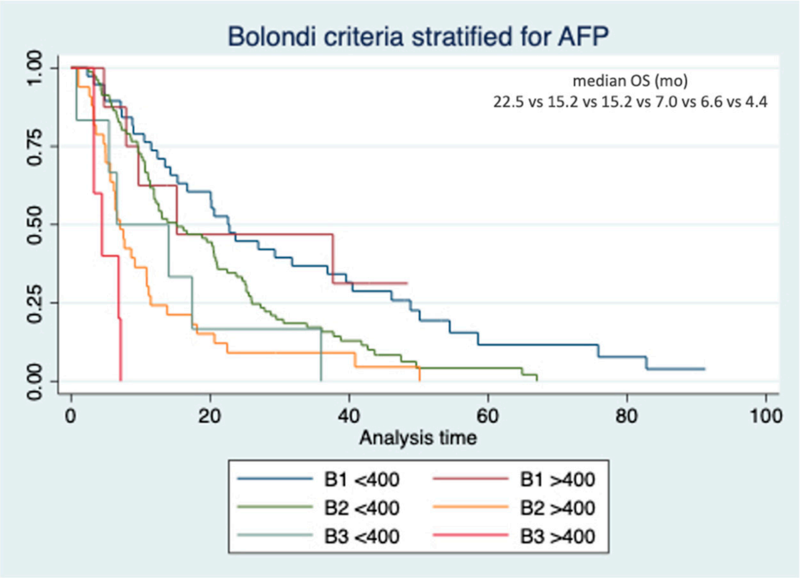

Supplementary Figure 1. Kaplan-Meier curves of overall survival according to Bolondi criteria stratified for alpha-fetoprotein.
